# Identification of the molecular subtypes and signatures to predict the prognosis, biological functions, and therapeutic response based on the anoikis‐related genes in colorectal cancer

**DOI:** 10.1002/cam4.7315

**Published:** 2024-05-24

**Authors:** Xiang Zhai, Baoxiang Chen, Heng Hu, Yanrong Deng, Yazhu Chen, Yuntian Hong, Xianghai Ren, Congqing Jiang

**Affiliations:** ^1^ Department of Colorectal and Anal Surgery Zhongnan Hospital of Wuhan University Wuhan China; ^2^ Clinical Center of Intestinal and Colorectal Diseases of Hubei Province (Zhongnan Hospital of Wuhan University) Wuhan China; ^3^ Hubei Key Laboratory of Intestinal and Colorectal Diseases (Zhongnan Hospital of Wuhan University) Wuhan China; ^4^ West China Hospital of Sichuan university Chengdu China

**Keywords:** anoikis‐related genes, extracellular matrix, TIMP1, tumor microenvironment

## Abstract

**Background:**

Tumors that resist anoikis, a programmed cell death triggered by detachment from the extracellular matrix, promote metastasis; however, the role of anoikis‐related genes (ARGs) in colorectal cancer (CRC) stratification, prognosis, and biological functions remains unclear.

**Methods:**

We obtained transcriptomic profiles of CRC and 27 ARGs from The Cancer Genome Atlas, the Gene Expression Omnibus, and MSigDB databases, respectively. CRC tissue samples were classified into two clusters based on the expression pattern of ARGs, and their functional differences were explored. Hub genes were screened using weighted gene co‐expression network analysis, univariate analysis, and least absolute selection and shrinkage operator analysis, and validated in cell lines, tissues, or the Human Protein Atlas database. We constructed an ARG‐risk model and nomogram to predict prognosis in patients with CRC, which was validated using an external cohort. Multifaceted landscapes, including stemness, tumor microenvironment (TME), immune landscape, and drug sensitivity, between high‐ and low‐risk groups were examined.

**Results:**

Patients with CRC were divided into C1 and C2 clusters. Cluster C1 exhibited higher TME scores, whereas cluster C2 had favorable outcomes and a higher stemness index. Eight upregulated hub ARGs (*TIMP1*, *P3H1*, *SPP1*, *HAMP*, *IFI30*, *ADAM8*, *ITGAX*, and *APOC1*) were utilized to construct the risk model. The qRT‐PCR, Western blotting, and immunohistochemistry results were consistent with those of the bioinformatics analysis. Patients with high risk exhibited worse overall survival (*p* < 0.01), increased stemness, TME, immune checkpoint expression, immune infiltration, tumor mutation burden, and drug susceptibility compared with the patients with low risk.

**Conclusion:**

Our results offer a novel CRC stratification based on ARGs and a risk‐scoring system that could predict the prognosis, stemness, TME, immunophenotypes, and drug susceptibility of patients with CRC, thereby improving their prognosis. This stratification may facilitate personalized therapies.

## 1 INTRODUCTION

Colorectal cancer (CRC) is the second most common cancer and the third leading cause of death worldwide.^1^ The incidence of CRC varies by up to 10‐fold in developing countries, with 2.5 million new cases worldwide predicted by 2035.^2^, ^3^ In China, the age‐standardized incidence rate and age‐standardized mortality of CRC rank second (23.90 per 100,000 population) and fifth (12.00 per 100,000 population), respectively,^4^ exerting considerable health and economic burdens. Significant progress has been made in CRC treatment, and current options include surgery, chemotherapy, and immunotherapy. However, CRC, as a typical solid cancer, involves multiple genomic mutations and instability, resulting in heterogeneity and different gene mutation subtypes,^5^ thereby leading to a heterogeneous response to chemotherapy and immunotherapy and a low overall 5‐year survival rate.^6^ Consequently, screening novel biomarkers to improve the clinical outcome of patients with CRC is crucial.

The extracellular matrix (ECM), composed of proteoglycans and fibrous proteins, is essential for cell and tissue differentiation and provides physical scaffolding and biochemical cues for cell evolution.^7^ Detachment of cells from the ECM triggers apoptosis, termed anoikis. Anoikis prevents the growth of incorrectly positioned cells, thus preventing tumor metastasis, and can be triggered by two main pathways: the death receptor pathway and the mitochondrial pathway.^8^ Detachment of cells by upregulating their death receptors, such as the FAS receptor, leads to the binding of the death ligands and activates the death receptor pathway to degrade special proteins and induce cell death. Conversely, the mitochondrial pathway is activated by mitochondrial damage and the secretion of factors such as cytochrome c, thereby inducing anoikis.^8^, ^9^, ^10^ However, malignant tumors can resist apoptosis, leading to metastasis.^11^, ^12^ As the downstream effector of Wingless‐related integration site signaling, transcription Factor 7 Like 2 (TCF7L2) is essential for regulating growth and metastasis in multiple cancers, such as lung cancer and CRC.^13^ TCF7L2 enhances anoikis resistance in gastric cancer,^14^ whereas cell migration‐inducing protein promotes anoikis resistance in prostate tumors by activating the miR‐1248/TM9SF4 axis.^15^ Cancer cells can also escape anoikis by regulating adhesion, such as protein‐L‐isoaspartate (D‐aspartate) O‐methyltransferase, which promotes the growth and metastasis of ovarian cancer by interacting with the ECM protein LAMB3.^16^ However, the molecular processes underlying anoikis resistance in CRC remain unclear.

Considerable progress has been made in treating advanced CRC using immunotherapy, an essential component of cancer treatment.^17^ However, immunotherapy approaches using immune checkpoint inhibitors (ICIs), such as PD‐L1 inhibitors and cytotoxic T lymphocyte antigen 4 (CTLA4) inhibitors, remain limited. The benefits of ICIs in patients with CRC with microsatellite stability (MSS) or mismatch repair proficiency (pMMR) are negligible.^18^ The tumor microenvironment (TME) is critical to immunotherapy resistance.^19^, ^20^ Myeloid‐derived suppressor cells, the components of the TME, can inhibit the activity of immune cells, such as effector T cells and natural killer cells.^20^ However, the mechanisms underlying the effect of anoikis genes on the TME, or immune infiltration, and the TME module remain unclear. Identifying different CRC subtypes based on anoikis gene profiles is pivotal for patient immunotherapy stratification and prognosis.

**FIGURE 1:**
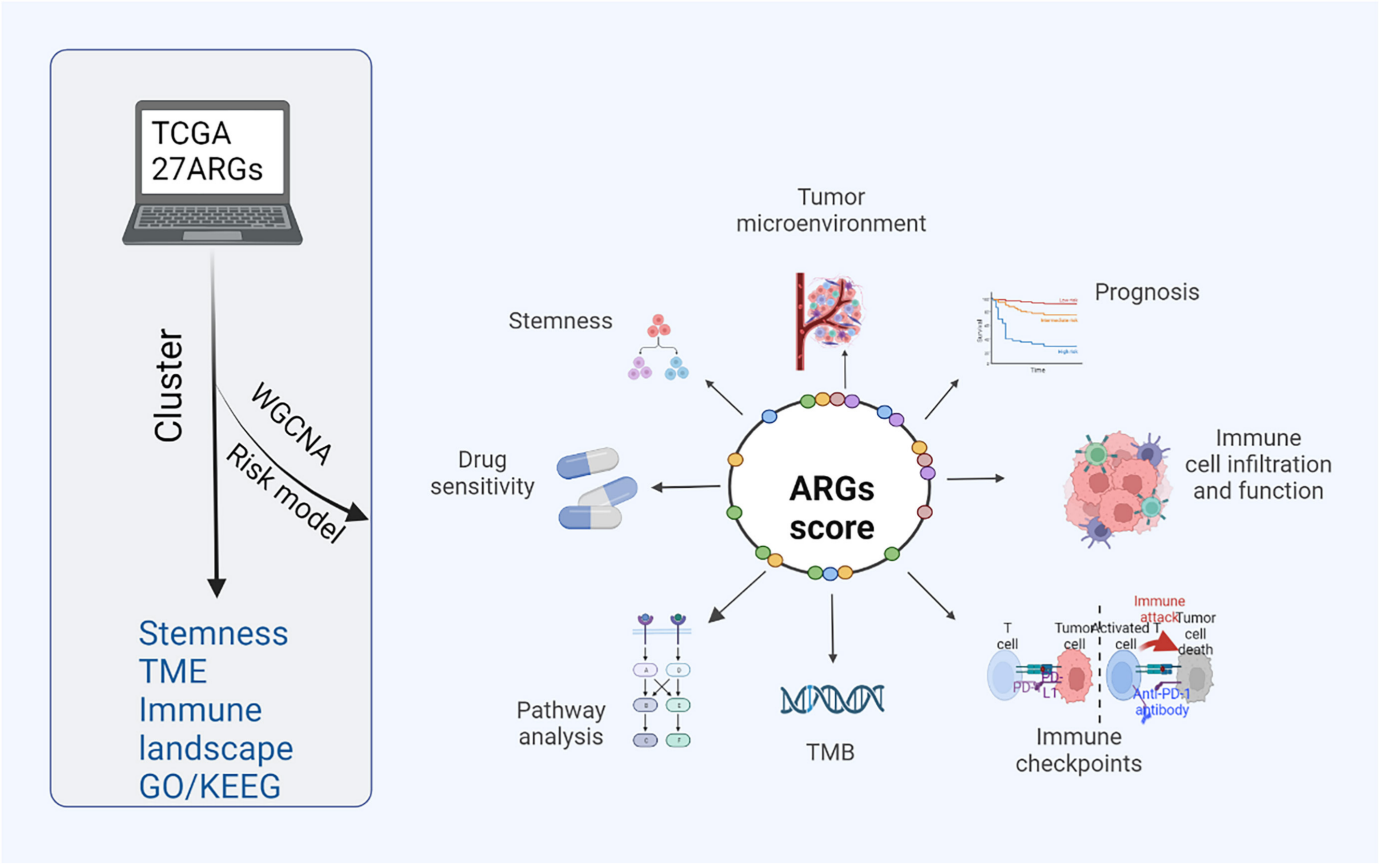
Study workflow, created with Biorender.com (https://www.biorender.com/). Permission of the copyright owner was obtained for publication.

In this study, we obtained gene expression profiles and anoikis‐related genes (ARGs) from public databases and conducted survival, stemness, TME, Kyoto Encyclopedia of Genes and Genomes (KEGG) pathway enrichment, and comprehensive immune landscape analyses to identify CRC subtypes. We also screened upregulated hub genes in CRC through weighted correlation network analysis (WGCNA) and Cox analysis. A risk model and prognostic nomogram were constructed based on eight genes validated using an external cohort. Finally, using a risk score model, we determined immune infiltration, immunotherapy response, drug sensitivity, and tumor mutation burden (TMB) (Figure 1). Screening and validating prognosis‐related ARGs may provide novel insights into the underlying biological processes of anoikis resistance in CRC and identify therapeutic targets, thereby facilitating the development of precision medicine.

## 2 MATERIALS AND METHODS

### 2.1 Data resources and preprocessing

The Cancer Genome Atlas (TCGA; https://portal.gdc.cancer.gov/) provides information on 620 transcripts per million (TPM) RNA sequencing (RNA‐seq) samples and matched CRC clinical data. All RNA‐seq data were transformed into log2 [TPM + 1]. A total of 27 ARGs were downloaded from the MsigDB database (http://www.gsea‐msigdb.org/gsea/index.jsp), all of which have been previously validated.^21^ The external validation cohort (GSE3882) and detailed clinical information were downloaded from the Gene Expression Omnibus (GEO) (https://www.ncbi.nlm.nih.gov/geo/). For multiple gene symbols, the average expression value was taken.

### 2.2 Somatic mutation analysis

We downloaded somatic mutation data from the Genomic Data Commons data portal of TCGA using the Mutation Annotation Format for patients with CRC. The “maftools” R package^22^ in R software was utilized to summarize and visualize the mutated genes.

### 2.3 Non‐negative matrix factorization (NMF) consensus clustering

The R package NMF (http://cran.r‐project.org/package=NMF) was used to perform NMF clustering.^23^ The NMF was run 10 times, and when both the consensus map and the cophenetic and silhouette coefficients were concurrently excellent, the number of clusters was 2. NMF clusters based on gene expression profiles were utilized to identify patient subgroups.^23^ Kaplan–Meier (KM) analysis was performed to plot survival curves. A log‐rank test was selected to compare the disease‐specific survival rates among the two risk groups.

### 2.4 Upregulated hub ARG extraction through WGCNA analysis and Venn diagram

The differentially expressed genes (DEGs) between two clusters were screened using the R package “limma”. WGCNA was used to screen the ARGs related to the TME in CRC. The differential expression‐related anoikis genes (DE‐ARGs) were concluded using the R package “edgeR” based on the false discovery rate<0.05 and |Log_2_FC| ≥ 1 and visualized by “ggplot2.” The “venndiagram” package intersected the ARGs related to the TME with upregulated DEGs.

### 2.5 Construction and validation of a novel prognostic model based on anoikis‐related genes

Using univariate Cox analysis, we identified survival‐associated genes. Utilizing the “glmnet” and “survival” R packages, we then developed a risk signature via least absolute selection and shrinkage operator (LASSO)–Cox regression.^24^ The formula is as follows:

$$\text{Risk score}=\sum_{j=1}^{n}\text{Coefj}*\mathrm{xj}$$

where *Xj* is the expression level of every prognostic ARG, and *Coef* is the coefficient.

Patients with CRC were categorized into low‐ and high‐risk groups based on the median scores. KM survival curves were used to compare overall survival (OS) and progression‐free survival (PFS) between the two groups. The “timeROC” R package was used to calculate the time‐dependent receiver operating characteristic (ROC) curve based on the signature's sensitivity and specificity (http://cran.r‐project.org/web/packages/timeROC).^25^ Furthermore, we divided TCGA samples into training and test sets to validate our model.

### 2.6 Construction of a prognostic nomogram

Using the R package “rms”,^26^ we constructed a predictive nomogram and corresponding calibration map based on the risk score and other critical clinical traits. The area under the curve (AUC) of the ROC was used to measure the diagnostic power of the nomogram.

### 2.7 Immune cell infiltration analysis

Various techniques, such as microenvironment cell population counters and CIBERSORT‐ABS, have been utilized to evaluate the makeup of immune cells infiltrating tumors. The “limma” package was used to investigate the association between cluster and immune cell infiltration risk levels. The Spearman correlation analysis from the “linkET” package assessed the relationship between risk scores, immune checkpoint‐related genes, immune cell infiltration, and immune functions.

### 2.8 Immunotherapy response predictions

Tumor immune dysfunction and exclusion (TIDE, http://tide.dfci.harvard.edu/) was used to assess the correlation between risk score and immune response.

### 2.9 Drug‐susceptibility evaluation

The immunophenoscores (IPS) of patients with CRC were obtained from The Cancer Immunome Database (TCIA, https://tcia.at/home). The Wilcoxon rank‐sum test was used to examine the connection between the IPS and risk signature. The “oncoPredict” program was used to forecast the drug sensitivity scores for CRC. In contrast, the “limma” package was used to analyze the relationship between the risk score and drug sensitivity score, which was visualized using the “ggplot2” package, to determine the therapeutic relevance of this risk model in CRC treatment.

### 2.10 Functional enrichment analysis

Gene Ontology (GO) and KEGG pathway enrichment analyses of ARGs were conducted using the R packages “limma” and “clusterProfiler”^27^ to identify the gene functions and biological pathways of the anoikis‐related DEGs. Next, we used gene sets from MSigDB version 4.0 and Gene Set Enrichment Analysis (GSEA) software (http://www.broadinstitute.org/gsea/index.jsp)^28^ to perform GSEA (c2.cp.kegg.v7.4. symbols.gmt). The gene set variation analysis (GSVA) R package was used to conduct GSVA enrichment analysis to further investigate the differences in biological function across the group.^29^

### 2.11 Cell culture

NCM460 (normal colonic epithelial cell line) and RKO, SW480, SW620, HCT116, HT29, and DLD1 (CRC cell lines) were purchased from the American Type Culture Collection and cultured with Dulbecco's Modified Eagle Medium supplemented with 10% fetal bovine serum and 1% penicillin G/streptomycin. The cells were maintained at 37°C in a humidified atmosphere with 5% CO_2_.^30^

### 2.12 Patient tissue samples of CRC

CRC tissue samples and matched normal tissues were obtained from patients undergoing surgery at Zhongnan Hospital, Wuhan, China, who had not previously received chemotherapy or radiotherapy. Each patient provided informed written consent for tissue collection. Tissues were immediately stored at ˗80°C.

### 2.13 RNA extraction and qRT‐PCR

Total RNA was extracted from the collected cells using the Trizol reagent (Invitrogen, USA) following the manufacturer's instructions for the All‐in‐One First‐Strand Synthesis Master Mix (Yugong Biotech, China), and cDNA was synthesized. Taq SYBR Green qPCR Premix was used to perform qRT‐PCR (Yugong Biotech) using the primers listed in Table S1. qRT‐PCR was performed in three biological replicates.

### 2.14 Western blotting

Radioimmunoprecipitation assay buffer (Biosharp, China) was used to extract the total protein from the cells and CRC tissues. Proteins were separated using 8%–12% SDS–PAGE and transferred onto polyvinylidene difluoride membranes. The membranes were blocked with quick‐block fluid and incubated with primary antibodies (Bioss, China), including TIMP1, overnight at 4°C. The bands were measured using an enhanced chemiluminescence substrate after being incubated for 2 h at room temperature with secondary antibodies.

### 2.15 Immunohistochemistry (IHC)

IHC was performed following the standard procedure. Briefly, the primary antibody TIMP1 polyclonal antibody (1:200, 16644‐1‐AP, Bioss, China) was added and incubated at 37°C overnight. The hematoxylin and eosin staining kit (Biosharp) was used to stain tissues.

### 2.16 Statistical analysis

R version 4.11 was used for data analysis. For nonparametric data, the Wilcoxon test was used for comparisons of two independent samples, whereas the Kruskal–Wallis test was used for multiple sample comparisons. For parametric data, an independent *t*‐test or one‐way ANOVA was performed. Spearman's correlation coefficients were used for all correlation analyses. R packages or Bioconductor packages were adopted, including the related R packages such as “survival,” “ggpubr,” “ggplot2,” “GSVA,” and “survminer.” *p* < 0.05 was considered statistically significant, and *p*‐values <0.01 and <0.001 were denoted by ** and ***, respectively.

## 3 RESULTS

### 3.1 Multiomic analysis of ARGs in CRC

**FIGURE 2:**
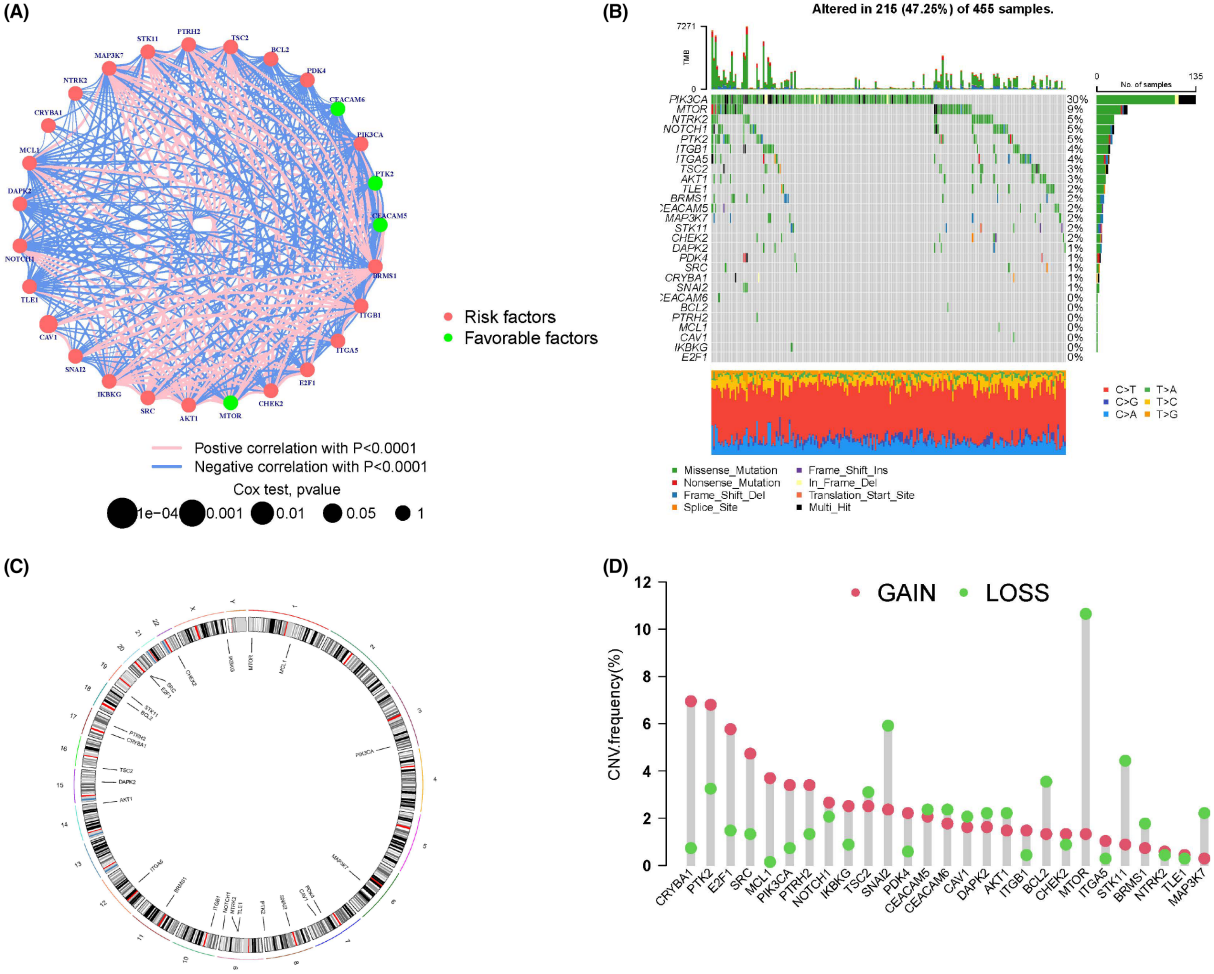
Multiomic analysis of ARGs in CRC. (A) Prognostic value analysis of 27 ARGs. (B). Mutation analysis of the 27 ARGs. (C) The circle plot exhibits the chromosomal distribution of 27 ARGs. (D) CNV frequencies of the 27 ARGs, red, green, and column height represent gain, loss, and frequency change, respectively.

We obtained 620 CRC samples from the TCGA database (Table S2) and downloaded 27 ARGs from the MSigDB.^21^ A network plot was used to display the comprehensive landscape of the correlation between ARGs and the prognostic value for CRC (Figure 2A; Figure S1A). We determined the somatic mutation prevalence of ARGs in CRC. Notably, PIK3CA had the highest mutation rate (up to 30%), followed by MTOR (9%), whereas other ARGs had lower mutation rates (Figure 2B). Furthermore, we identified the CNV alterations in 27 ARGs (Figure 2C) and observed that CNV‐related mutations in these ARGs were common in CRC. Specifically, *CRYBA1*, *E2F1*, *SRC*, *MCL1*, *PIK3CA*, *PTRH2*, and *PTK2* exhibited extensive CNV amplification, whereas *MTOR*, *SNAI2*, *STK11*, *MAP3K7*, and *BCL2* exhibited CNV deletions (Figure 2D).

### 3.2 Traits of stemness, TME, immune landscape, and pathway enrichment analysis of clusters

**FIGURE 3:**
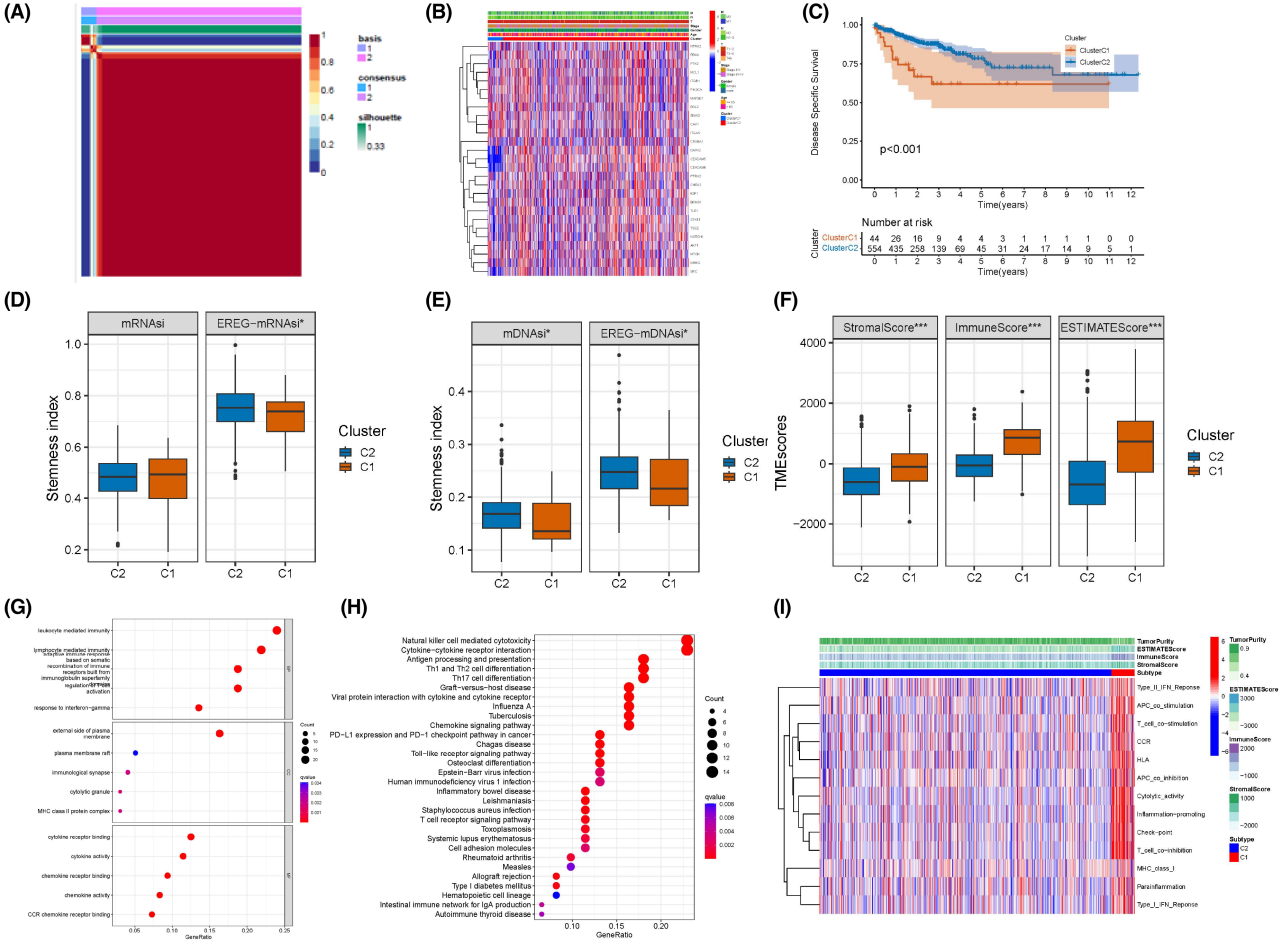
Extensive analysis of ARGs biological traits. (A) Consensus clustering matrix (*k* = 2). (B) The heatmap shows the clinical traits of C1 and C2 clusters. (C) KM curve demonstrates the survival difference of the two clusters, *p* < 0.01. (D, E). Boxplots shows differences in stemness between the two clusters. (F) The boxplot shows differences in TME between the two clusters. (G, H) GO and KEEG analysis between two subgroups. (I) The heatmap exhibits the immune landscape of the two clusters.

We classified patients with CRC into C1 and C2 clusters using NMF consensus clustering based on the ARG expression levels (Figure 3A; Figure S1B). The relationship between the clinical characteristics of patients with CRC (age, sex, and T, N, and M stages) and clusters is shown in Figure 3B and Table S3. The KM curve demonstrated that patients in cluster C2 had significantly better clinical outcomes than those in cluster C1 (Figure 3C; *p* < 0.001, log‐rank). A stemness index analysis was conducted to assess the differences in stemness between the C1 and C2 clusters. Figure 3D–F displays the stemness of gene expression levels; EREG‐mRNAsi and the methylation stemness index (mDNAsi and EREG‐mDNAsi) of the C2 cluster were higher than those of the C1 cluster (Figure 3D,E). Considering the different clinical outcomes of the two clusters, we explored the biological differences, including TME, immune cell infiltration, and immune function. The C1 cluster exhibited a higher TME score, including stromal, immune, and estimate scores (Figure 3F; *p* < 0.001), and infiltration of activated monocytes, M1 macrophages, and mast cells, than the C2 cluster. Meanwhile, cluster C2 showed more significant infiltration of M0 macrophages and activated mast cells (Figure S1C). To explore the potential biological functions of ARGs, GO analysis was conducted, which indicated that ARGs were correlated with immunity, including leukocyte‐mediated immunity and lymphocyte‐mediated adaptive immune responses. ARGs were also associated with cytokine receptor binding and the external side of the plasma membrane (Figure 3G). KEGG pathway analysis revealed that natural killer cell‐mediated cytotoxicity, cytokine–cytokine receptor interactions, antigen processing and presentation, PD‐L1 expression, the PD‐1 checkpoint pathway in cancer, and the T cell receptor signaling pathway were significantly associated with ARGs (Figure 3H). Figure 3I presents an overview of the relationship between the clusters, TME, and immune function.

### 3.3 Construction and validation of ARG prognostic signatures in CRC

**FIGURE 4:**
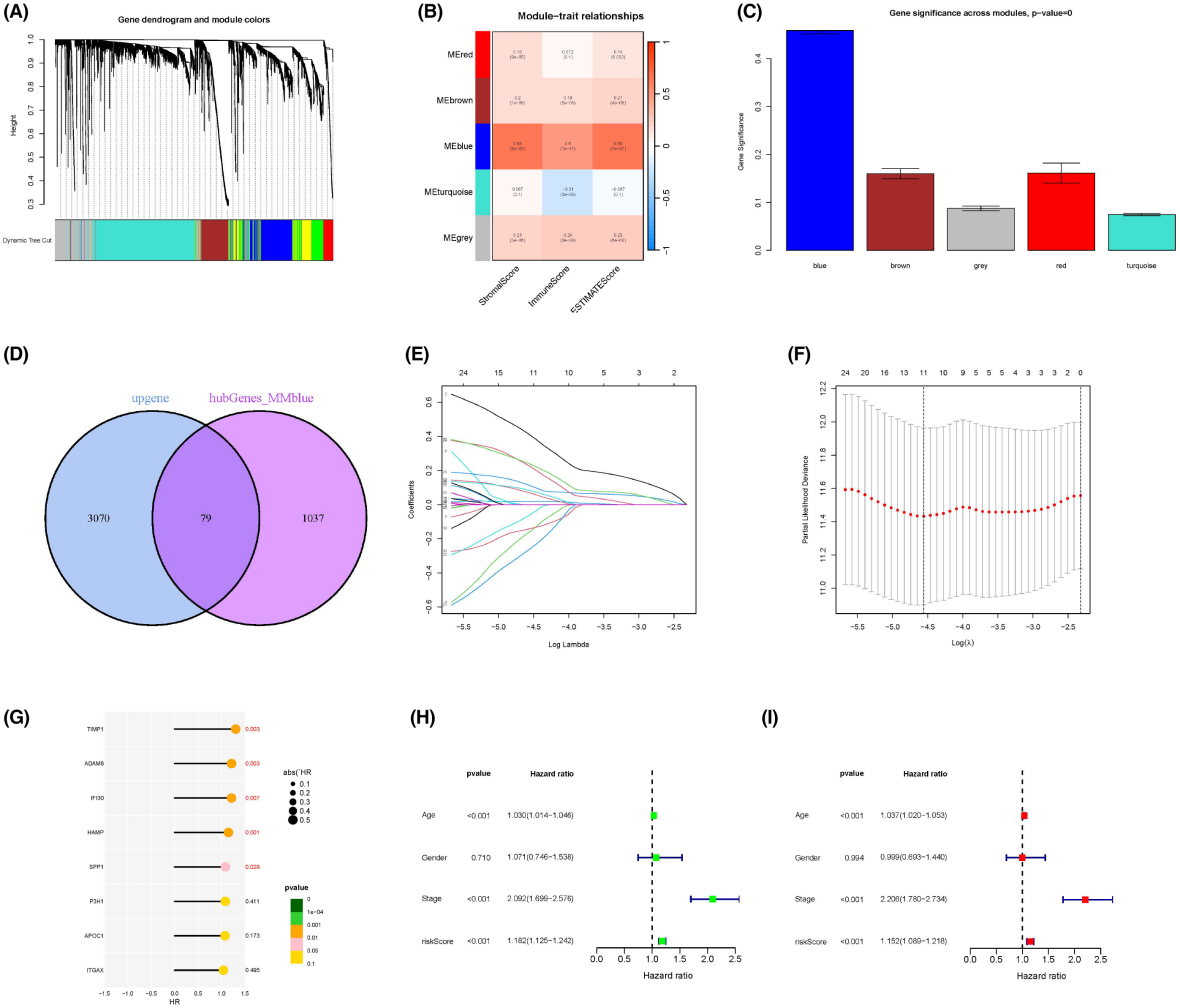
Constructing the risk model through WGCNA and the LASSO regression algorithm. (A) Clustering dendrogram showing that the genes with dissimilarity based on topological overlap with the related module colors. (B) The heatmap showed the association between module eigengenes and TME scores. (C) Column of gene significance module. (D) Venn diagram of upregulated genes and hub‐blue module. (D, E) LASSO analysis of ARGs associated with prognosis. (G) Risk of eight ARGs predicting survival and prognosis in patients with CRC. (H, I) KM curve of OS (*p* < 0.01) and PFS (*p* < 0.01) in all sets. (J, K) Univariate and multivariate regression analysis of clinical traits and risk score. (L) ROC curve of risk score, nomogram, and clinical features.

To screen the hub ARGs, 5314 DEGs between the two clusters (Figure S2A) were included in the WGCNA analysis in the CRC cohort. The soft threshold power was 9, and dynamic module identification was performed with >50 genes per module (Figure 4A; Figure S2B). The highest relationships with the clinical traits were observed in the MEblue (cor = 0.42, *p* = 9.8e−79, cor = 0.71, *p* < 1e−200; Figure 4B,C; Figure S2C,D). Previous studies have demonstrated the involvement of the upregulated genes in the tumorigenicity of multiple cancers and suggested their potential application in diagnosis. For example, upregulated genes in CRC are associated with transcriptional cis‐effects of copy number gains.^31^ In contrast, upregulation of lipid metabolism genes has been shown to promote the development of breast cancer and can be applied in early diagnosis.^32^ Therefore, we focused on the upregulated genes in the CRC. In total, 1097 hub genes in the MEblue model and 3070 upregulated genes were intersected, which were used to the univariate analysis (Figure 4D; Figure S2E,F). Prognostic genes were used for the LASSO regression algorithm to construct the risk model. Eight upregulated ARGs were obtained in our risk score model, and the following risk formula was used: risk score = TIMP1 × (0.76644) + P3H1 × (˗0.58726) + SPP1 × (0.20091) + HAMP × (0.29253) + IFI30 × (0.52407) + ADAM8 × (0.43338) + APOC1 × (˗0.33346) + ITGAX × (˗0.67368) (Figure 4E–G; Table S4; Figure S3A–H). The expression pattern was validated through the IHC of those genes, except for HAMP, from the HPA database (Figure S6). KM survival curves were used to compare OS and PFS between the high‐ and low‐risk groups in the train, test, and all sets. The high‐risk group had significantly worse outcomes (all *p*‐values < 0.01; Figure 4H,I; Figure S2C–F). In addition, univariate and multi‐univariate Cox regression algorithms for the risk score and clinical traits indicated that risk score and stage were independent adverse prognostic factors (Figure 4J, K; Figure S4). The AUC of the ROC for the risk score in the train, test, and all sets was 0.658, 0.611, and 0.730, respectively, indicating that our risk model had good prediction ability (Figure 4L; Figure S2G,H).

### 3.4 Construction and estimation of the prognostic prediction nomogram

**FIGURE 5:**
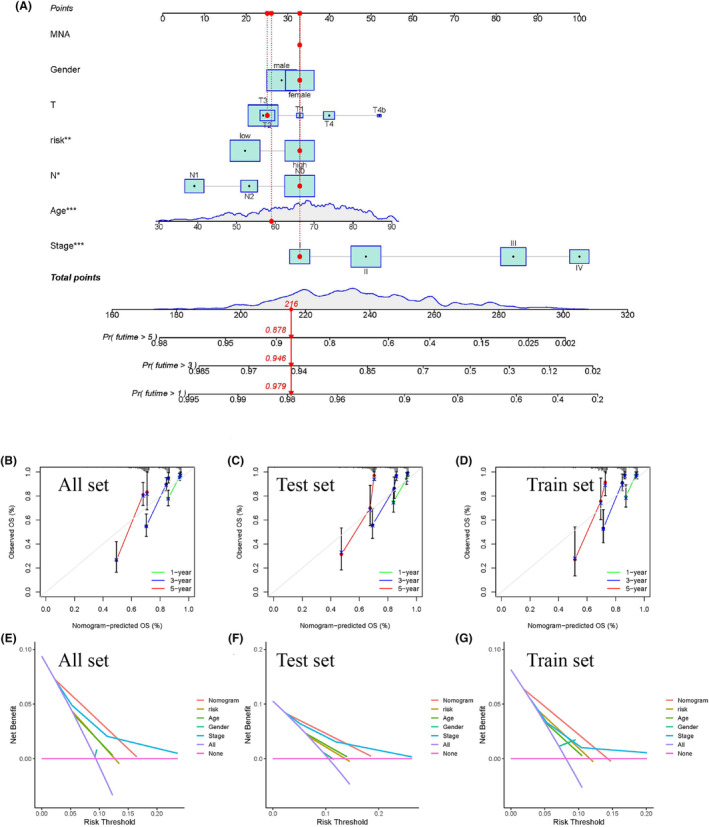
Nomogram construction (A) Nomogram diagram that integrated the risk score and clinical features. (B–D) Verification and assessment of the ROC of the three sets containing nomogram. (E–G) DCA of three sets containing nomogram.

To assess the prognostic value of our risk model in patients with CRC, a 1‐, 3‐, and 5‐year OS predictive nomogram and a corresponding calibration map based on the risk score and clinical features were constructed (Figure 5A). The nomogram's discriminatory power and clinical usefulness were estimated using calibration and ROC curves. Calibration plots for the 1‐, 3‐, and 5‐year OS rates were based on the adjusted line (Figure 5B–D). The AUC of the ROC curve for the nomogram and stage in all sets (0.803, 0.714), the test set (0.828, 0.744), and the training set (0.730, 0.670) showed favorable predicted performance in our model (Figure 4L; Figure S2G,H). Further verification and assessment of the three sets and their decision curve analysis (DCA) contained a nomogram (Figure 5B–G).

### 3.5 Relationship between risk score and stemness, TME, immune traits, and functional and pathway enrichment

**FIGURE 6:**
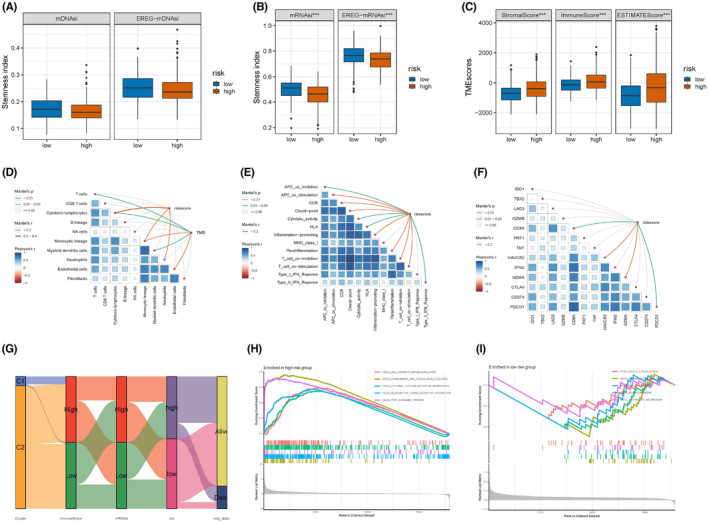
Distinct biologic characteristics of the two risk subgroups. (A, B) The boxplot showed the stemness differences between the two risk groups. (C) TME analysis of the two risk groups. (D–F) Immune cell infiltration, immune function, and immune checkpoints of the two risk subgroups. (G) Overview of the correlation of cluster, risk score, stemness, TME, and survival status. (H, I) Functional analysis by GSEA.

As TME and immune infiltration were associated with immunotherapy resistance in patients with CRC, we explored the TME between the two risk groups. ESTIMATE analysis of the TME demonstrated that patients with a high‐risk score had significantly higher stromal (*p* < 0.01), estimate (*p* < 0.01), and immune (*p* < 0.01) scores than those with a low‐risk score (Figure 6C). The relationship of different immune cell infiltrations between the two subgroups showed high similarity, except for resting mast cells (Figure 6D; Figure S5A). Immune activity significantly differed between the two risk groups, including type II or I, IFN response, APC co‐stimulation or co‐inhibition, cytolysis, CCR, HLA, inflammation‐promoting, immune checkpoint, and T‐cell co‐inhibition, indicating that the high‐risk group had a worse prognosis and benefited from immunotherapy (Figure 6E). We explored the immune checkpoints associated with the risk score and obtained favorable results. Patients with a high‐risk score exhibited higher immune checkpoint levels, such as PD‐L1 (*p* < 0.01), CTLA4 (*p* < 0.01), PDCD1 (*p* < 0.01), and TNF (*p* < 0.01) (Figure 6F). These results may help facilitate immunotherapy in CRC. An overview of the cluster, risk score, stemness, TME, immune score, and survival status is presented in Figure 6G.

The results demonstrated that clinical traits, survival status, and immune landscapes were associated with the risk score; therefore, we hypothesized differences in the underlying biological mechanisms of the two subgroups. Consequently, we conducted a GSEA analysis. Figure 6H shows that the PPAR signaling pathway, an essential target of human metabolism‐related diseases, including cancers, was enriched in the high‐risk subgroups. Meanwhile, cell adhesion molecules related to cell–cell or cell–ECM adhesion were significantly enriched in the high‐risk subgroup. The complement and coagulation cascades, cytokine receptor interaction, and neuroactive ligand–receptor interaction were significantly enriched in the high‐risk group. Meanwhile, the citrate cycle (TCA cycle), DNA replication, and base excision repair were significantly enriched in the low‐risk subgroup. Interestingly, the mismatch repair pathway, associated with MSS and microsatellite instability (MSI), was significantly enriched in the low‐risk group (Figure 6I).

### 3.6 MSI and TIDE analysis between high‐ and low‐risk groups

**FIGURE 7:**
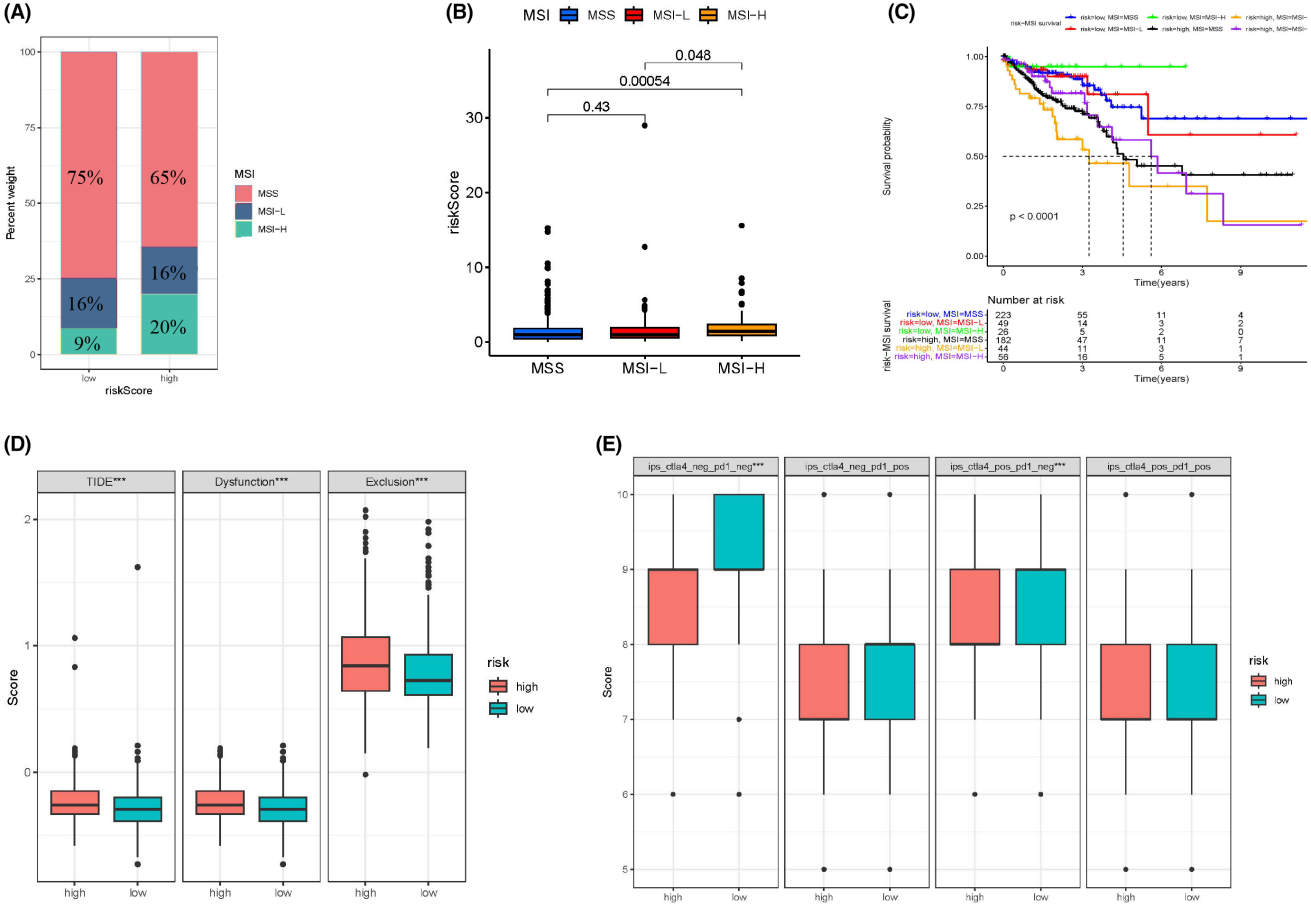
MSI, TIDE, and immune response analysis between the two risk groups. (A, B) MSI status between the different risk groups. (C) The KM curve showed that risk high‐MSI‐H harbored the worst clinical outcome (*p* < 0.01). (D) TIDE score analysis of risk groups. (E) Immunotherapy response analysis in different groups.

MSI status affects the potency of PD‐1 or PD‐L1 inhibitors in CRC; therefore, we investigated the correlation between the MSI status and risk score and identified an association between the percentage of MSI‐H and the risk score. MSI‐H in the high‐risk subgroup accounted for 20% of CRC cases, significantly higher than that in the low‐risk subgroup (9%) (Figure 7A,B; Figure S5E).

Moreover, we determined whether survival status was associated with MSI status and risk score (Figure 7C) and investigated immunotherapy and immune escape in the different risk groups. TIDE score analysis showed that patients with CRC in the high‐risk subgroup had higher T cell dysfunction or exclusion scores than those in the low‐risk subgroup (Figure 7D). However, no differences were observed in the immunotherapy analysis between the high‐ and low‐risk subgroups, except for ips_ctla4_neg_pd1_neg (*p* < 0.01) (Figure 7E).

### 3.7 Mutations and drug‐susceptibility analysis

**FIGURE 8:**
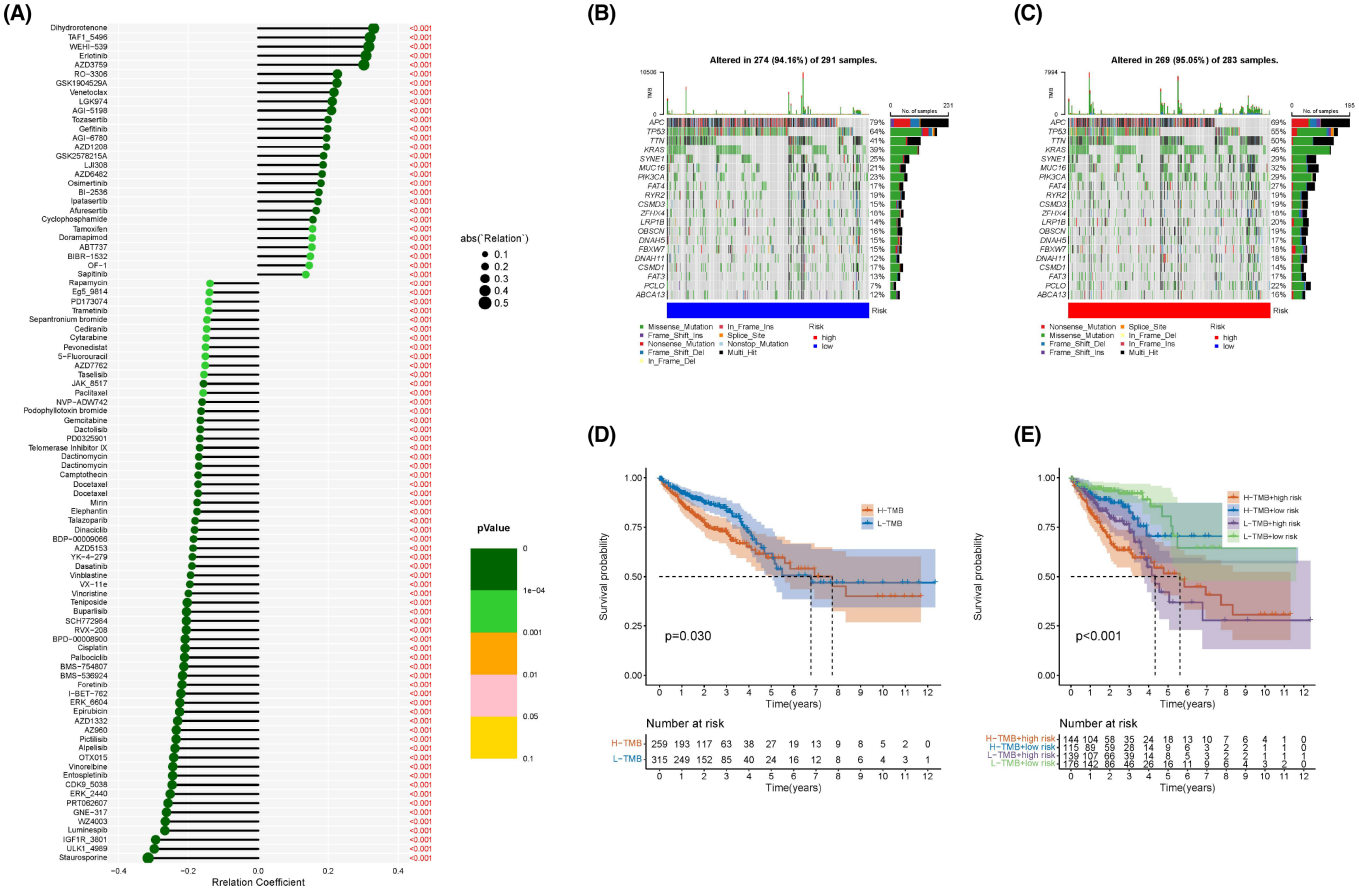
Mutation profiles and drug sensitivity analysis of two risk groups. (A) drug‐susceptibility analysis between high‐ and low‐risk group, colors and black circle represent the *p*‐values and correlation coefficients, respectively. (B, C) Waterfall plot of the different risk groups with red and blue representing the high‐ and low‐risk group, respectively. (D, E) KM curve of H‐and low‐TMB or associated with risk score (*p* < 0.05, 0.01, respectively).

In typical solid cancers, somatic mutations, to some extent, are critical factors of tumor occurrence and development and are therefore potential biomarkers of targeted chemotherapy. Figure 8B shows that TMB in the high‐risk subgroup was higher than in the low‐risk group. The mutation rates of oncogenes *KRAS*, *MUC16*, *PIK3CA*, *FAT4*, and *PCLO* were significantly higher in the high‐risk subgroup than in the low‐risk group, whereas the tumor suppressor genes *APC* and *TP53* were lower in the high‐risk subgroup (Figure 8C). Furthermore, the KM curve demonstrated that a low TMB was associated with a better clinical outcome (*p* < 0.030), particularly in patients with low‐risk CRC (*p* < 0.01) (Figure 8D,E). Accordingly, we investigated the potential of using the risk score to guide targeted chemotherapy and molecular targeted therapy. Drug susceptibility (IC_50_ values) showed that patients in the high‐risk group were more sensitive to drugs such as stauroporine, ERK_2440, alpelisib, cisplatin, 5‐fluorouracil, and entospletinib. Patients with a high‐risk score were more sensitive to dihydrorotenone, taf15496, eriotinib, AZD3759, gefitinib, and osimertinib (Figure 8A). These results demonstrated that, based on the present ARGs, the risk score can help facilitate precise CRC chemotherapy or molecular‐targeted therapy and provide personalized treatment.

### 3.8 External verification of risk score using GEO cohort

**FIGURE 9:**
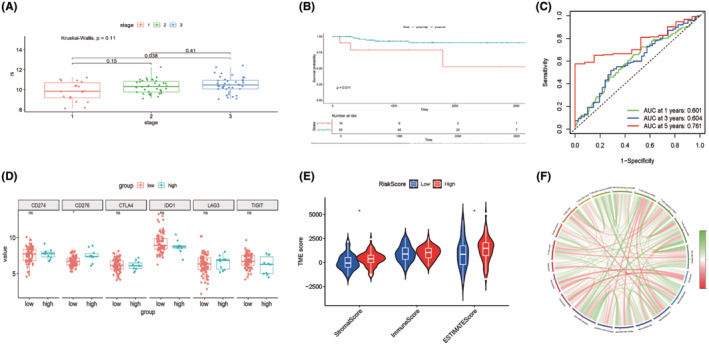
External verification of risk score using GEO cohort (A) The relationship of risk score and CRC stage. (B) KM curve of anoikis‐based score in external cohort. (C) ROC analysis of anoikis‐based score. (D) The correlation of immune checkpoints and anoikis‐based score. (E) The relationship of TME score and anoikis‐based score. (F) The correlation of immune infiltration and anoikis‐based score.

We used the external GEO cohort to validate the reliability of the anoikis‐based risk model. Patients with CRC from the GEO cohort (GSE3882) were divided into two risk groups using the risk score formula. Consistent with the results of the TCGA dataset, patients with stage 3 CRC had higher risk scores (Figure 9A), and the high‐anoikis score group showed worse prognoses than the low‐anoikis score group (Figure 9B). Moreover, the ROC revealed AUC values of 0.601, 0.604, and 0.761 for predicting the 1‐, 3‐, and 5‐year OS, respectively (Figure 9C). Beneficial effects in patients with advanced CRC have been achieved using ICIs due to the activation of the immune response, particularly by blocking the combination of activated T cells and PD‐L1, which otherwise compromises the immunity in patients with CRC.^33^, ^34^ To explore the possibility of anoikis‐based score‐guided precise immunotherapy, we supplemented the analysis of the anoikis‐based score and immune checkpoints. We found that the high‐risk group of the GEO cohort showed higher expression levels of PD‐L1 (Figure 9D). We also estimated the relationship between the anoikis‐based score and immune infiltration cells in the external cohort and showed similar immune infiltration levels in the TCGA cohort (Figure 9F).

### 3.9 External verification of the expression patterns of risk signature genes

**FIGURE 10:**
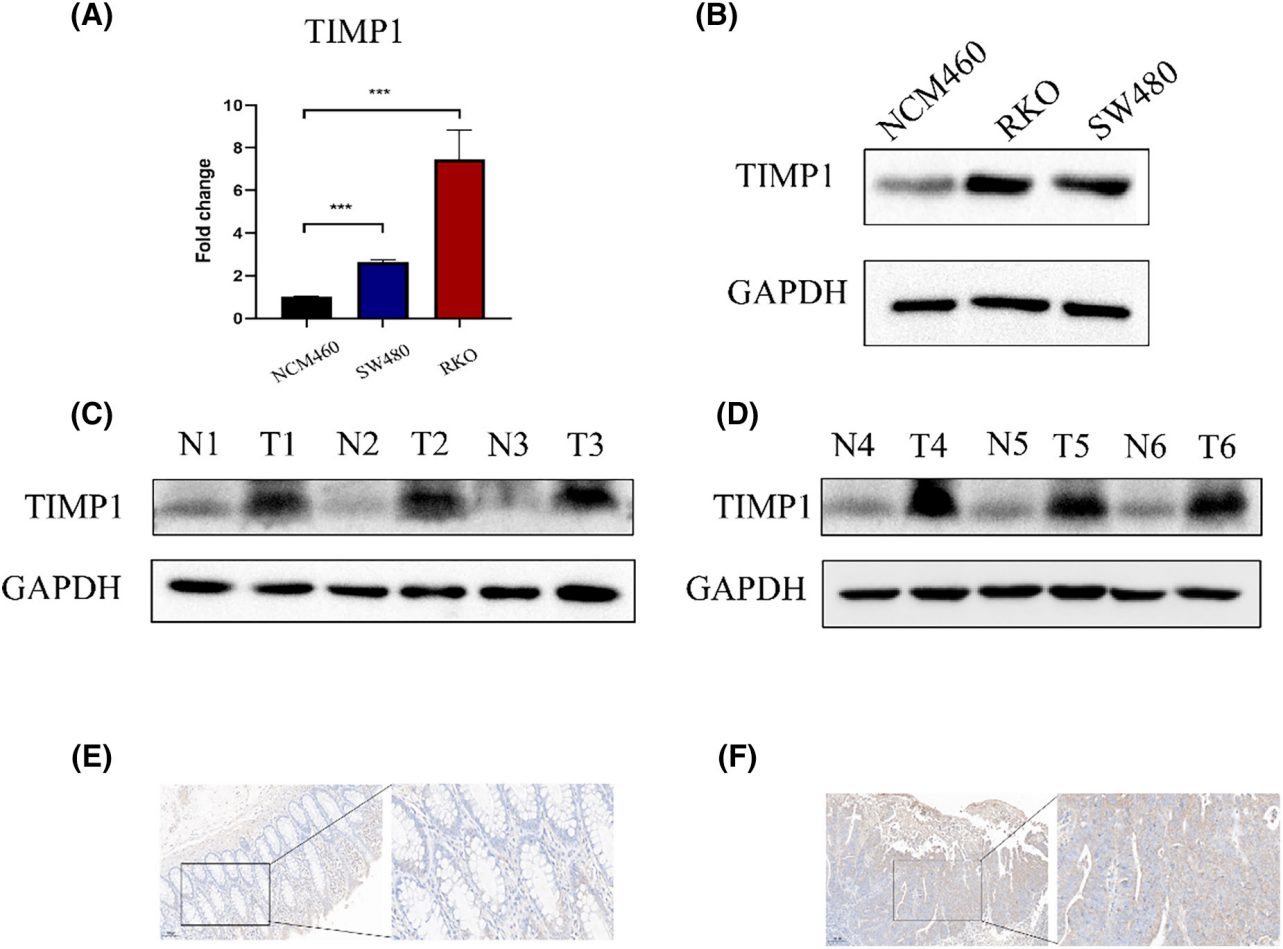
External validation of TIMP1 expression. (A) qRT‐PCR of NCM460 and CRC cell lines (***p < 0.001). (B) Protein expression of CRC cell lines and normal colonic epithelial. (C, D) Protein expression of TIMP1 in six paired CRC tissues, (E, F) Immunohistochemistry of TIMP1 in CRC tissues, E and F represented normal and CRC tissue, respectively.

Common CRC cell lines (DLD1, HCT116, HT29, SW480, SW620, and RKO) and the normal colonic epithelial cell line NCM460 were used to validate the expression patterns of hub genes. qRT‐PCR demonstrated that the expression of hub genes was upregulated in CRC cells (Figure S7). Specifically, *TIMP1* was upregulated in SW480 and RKO cells (approximately 3 and 7 times, respectively) (Figure 10A). Due to the highest coefficient value of TIMP1 in the risk model, we selected TIMP1 for further validation. The protein expression showed the same pattern (Figure 10B). CRC tissues and paired normal tissues (clinical characteristics are listed in Table S5) were collected, and differential expression of the ARG signature TIMP1 was confirmed by tissue protein or IHC (Figure 10C–F).

## 4 DISCUSSION

Anoikis, the apoptosis of cells in abnormal locations, is primarily triggered by the death receptor and mitochondrial pathways and plays a significant role in preventing anomalous proliferation of cells, thereby decreasing cancer metastasis.^8^, ^35^ However, tumors can escape anoikis through molecular changes, such as overexpression of FILP in malignant cells or downregulation during cell detachment, aberrant lncRNA expression, or Bcl 2 family protein.^10^, ^36^ Previous studies have demonstrated that overcoming anoikis resistance mitigates drug resistance and prevents cancer proliferation and metastasis. For example, TCF7L2 downregulation inhibits gastric cancer cell growth and promotes apoptosis^14^; however, the correlation between anoikis resistance and TME, immune landscape, and TMB, particularly in CRC, remains unclear.

In the present study, we estimated the role of 27 ARGs in CRC, including stemness, somatic mutation, and immune landscape. Our results showed that *KRAS* and *PIC3CA* mutations were more prevalent in the high‐risk group than in the low‐risk group, whereas the low‐risk group exhibited more *TP53* mutations. Mathieu et al.^37^ demonstrated that a *KRAS* mutation was essential to maintaining tumorigenicity by promoting anoikis resistance in CRC, which is consistent with our findings. *PIK3CA* is an oncogene involved in several cancers, including breast cancer, gastric cancer, and CRC. The breast cancer cell lines E545K and H1047R, which harbor *PIK3CA* mutations, exhibited anoikis resistance and promoted tumorigenicity.^38^ Furthermore, we classified patients with CRC into two clusters and found that patients in the C1 cluster had worse clinical outcomes yet a higher immune score; therefore, we examined the DEGs between the two clusters and conducted WGCNA analysis. Eight hub ARGs (*TIMP1*, *P3H1*, *SPP1*, *HAMP*, *IFI30*, *ADAM8*, *ITGAX*, and *APOC1*) upregulated in CRC were identified through WGCNA and LASSO analyses.

The expression patterns of the hub genes were validated using qRT‐PCR, Western blotting, or IHC, which yielded consistent results with those of bioinformatics analysis. TIMP1 is an inhibitor of the metalloproteinases family and confers anoikis resistance in melanoma.^39^ In CRC, TIMP1 is an independent prognostic indicator.^40^ Furthermore, TIMP1 promotes CRC proliferation and metastasis through the FAK‐PI3K/AKT and MAPK pathways.^41^ SPP1 was upregulated in CRC and promoted CRC growth and immune infiltration, particularly of SPP1^+^ macrophages and CD8^+^ T cells. Furthermore, *SPP1* is an anoikis gene in idiopathic pulmonary fibrosis.^42^, ^43^ To the best of our knowledge, the roles of *P3H1*, *HAMP*, *IFI30*, *ADAM8*, *ITGAX*, and *APOC1* in anoikis have not been reported.

Based on the screened ARGs, we constructed an ARG risk score model. Notably, the ARG risk model can be an independent prognostic factor for CRC. Cai et al.^44^ divided CRC samples into high‐ and low‐risk groups based on the ARG score determined using the “survminer” R package and screened three signatures to predict CRC prognosis. Other studies have also constructed a prognostic model for CRC.^45^, ^46^ In the present study, we screened the prognostic signatures for CRC and explored the correlations with clinical characteristics, stemness, TME, TMB, immune filtration, and drug susceptibility to determine the role of anoikis genes in CRC. Moreover, the expression patterns of hub genes were validated using in vitro experiments. Simultaneously, the anoikis‐risk model was verified using an external GEO cohort, confirming the reliability of our model.

Beneficial effects in patients with advanced CRC have been achieved using ICIs due to the activation of the immune response, particularly by blocking the combination of activated T cells and PD‐L1, which otherwise compromises the immunity in patients with CRC.^33^, ^34^ Nevertheless, the clinical outcome of ICIs in patients with advanced CRC remains unsatisfactory owing to congenital ICI resistance or no response to immunotherapy. Notably, ICI resistance is significantly associated with microbiota and tumor‐intrinsic heterogeneity (MHC degradation and chromatin remodeling), particularly in the TME.^19^, ^33^, ^47^ Therefore, targeting TME is a potential strategy for enhancing the immune response to ICIs in patients with advanced CRC. In our model, we drew a TME landscape in patients with CRC and investigated the correlation with the risk score. The stromal and immune scores were higher in the high‐risk subgroup than in the low‐risk subgroup. Therefore, the TME in patients with CRC is diverse and associated with the ARG risk score. Stromal cells, ECM, infiltrating immune cells, and cytokines are essential components of the TME and contribute to regulating tumor biological characteristics such as growth and metastasis.^48^ For example, tumor‐stromal interaction, which is associated with tumorigenicity in CRC, can be significantly enhanced by TGF‐β activation in TME.^49^ Daniele et al.^50^ observed that high levels of TGF‐β in TME in CRC induced a weak immune response and that blocked TGF‐β in TME could enhance the effect of PD‐1 or PD‐L1 immunotherapy and decrease metastasis in a mouse model. The function of CD8^+^ T immune cells can be disabled by immunosuppressive cytokines in the TME.^47^ In this study, we proposed a novel TME classification based on the ARG risk score, which may contribute to the development of novel personalized immunotherapy regimens and improve prognosis in patients with CRC. Moreover, immune checkpoint analysis between the two subgroups revealed that TNF, CTLA4, PDCD1, CD8, and PD‐L1 expression was more enriched in the low‐risk subgroup than in the high‐risk subgroup. Inhibition of CTLA4 can improve the immune response of T cells and is a significant target of immunotherapy.^51^ CTLA4 inhibitors may interact with T regulatory cells in the TME under specific conditions.^52^ These results provide a new molecular stratification of patients with CRC and will facilitate the development of personalized immunotherapy.

Despite progress in immunotherapy, some limitations remain, particularly in patients with CRC with MSS and MSI‐L, which are obstacles to successful immunotherapy with PD‐L1 inhibitors.^17^ We explored the MSI status discrepancy based on the risk score, which revealed that the percentage of MSI‐H in the high‐risk subgroup was significantly higher than in the low‐risk group. Importantly, patients with high‐risk MSI‐H exhibited the poorest clinical outcomes. Our pathway enrichment analysis revealed discrepant enrichment in different risk groups, such as the mismatch repair pathway. Several studies have demonstrated that immune cell infiltration in dMMR and pMMR varies significantly.^33^, ^51^ Pelka^53^ conducted a transcriptional profile analysis of both pMMR and dMMR CRC tissues and showed that dMMR enriched the activated T cells and T cell‐enrolling chemokines. Although no significant differences were observed in immune cell infiltration, our findings (immune function: cytolytic activity, APC co‐inhibition, type I or II INF response [Figure 6D,E] or ICI expression: CTLA4, PD‐L1, LAG3, TNF [Figure 6F]) are consistent with those reported by Pelka. Furthermore, immune response analysis (PD1 and CTLA4 analyses) demonstrated that patients in the low‐risk group could benefit more from ips_ctla4_neg_pd1_neg or ips_ctla4_pos_pd1_neg, and TIDE analysis also supported this finding.

This study has some limitations. The public database (TCGA) provided the transcriptome profiles of CRC, and the analytical method NMF significantly increased bias. Furthermore, the hub gene expression patterns were confirmed in vitro, and CRC tissues were not verified for any hub genes other than *TIMP1*. In subsequent research, we intend to collaborate with the Hubei Key Laboratory of Intestinal and Colorectal Diseases and the Hubei Provincial Human Genetic Resources Collection Center to construct a dataset for classification validation. Lastly, although the risk score was significantly correlated with TME, MMR status, and prognosis, the underlying molecular process was not investigated. In the future, we will examine the specific molecular activities of the ARGs or ARG score in TME or MMR status, which will help to develop anoikis‐based target treatments.

## 5 CONCLUSION

We screened upregulated ARGs in CRC gene sets and constructed a risk model based on their expression. Our results demonstrated that ARG risk scores were significantly associated with clinicopathological traits, stemness, TME, immunological phenotypes, and therapeutic targets in CRC, providing a prognostic prediction model for patients with CRC and allowing treatment based on patient stratification.

## AUTHOR CONTRIBUTIONS

**Xiang Zhai:** Conceptualization (lead); data curation (equal); formal analysis (equal); methodology (equal); writing – original draft (equal). **Baoxiang Chen:** Conceptualization (equal); data curation (equal); investigation (equal). **Heng Hu:** Data curation (equal); writing – original draft (equal). **Yanrong Deng:** Data curation (equal); writing – original draft (equal). **Yazhu Chen:** Data curation (equal). **Yuntian Hong:** Data curation (equal); formal analysis (equal); writing – original draft (equal). **Xianghai Ren:** Conceptualization (equal); formal analysis (supporting); supervision (supporting). **Congqing Jiang:** Methodology (supporting); writing – original draft (equal); writing – review and editing (lead).

## FUNDING INFORMATION

This study was funded by Zhongnan Hospital of Wuhan University Medical Science and Technology Innovation Platform Construction Support Project, No. PTXM2023028.

## CONFLICT OF INTEREST STATEMENT

There are no conflicts of interest to declare.

## ETHICS STATEMENT

The study protocol was approved by the Ethical Committee of Zhongnan Hospital of Wuhan University. All patients provided written informed consent to participate in the study.

## Supporting information


Appendix S1.

## Data Availability

All data were downloaded from the public database, and CRC tissue and paired clinical traits were obtained from Zhongnan Hospital of Wuhan University, China.
