# Supplementary material for: Identification of the molecular subtypes and signatures to predict the prognosis, biological functions, and therapeutic response based on the anoikis‐related genes in colorectal cancer
Source: Cancer Med. 2024 May 24;13(10):e7315. doi: 10.1002/cam4.7315 (PMC11117457; doi:10.1002/cam4.7315)
Supplement: Supplementary file 1 — Appendix S1. [file CAM4-13-e7315-s001.docx]

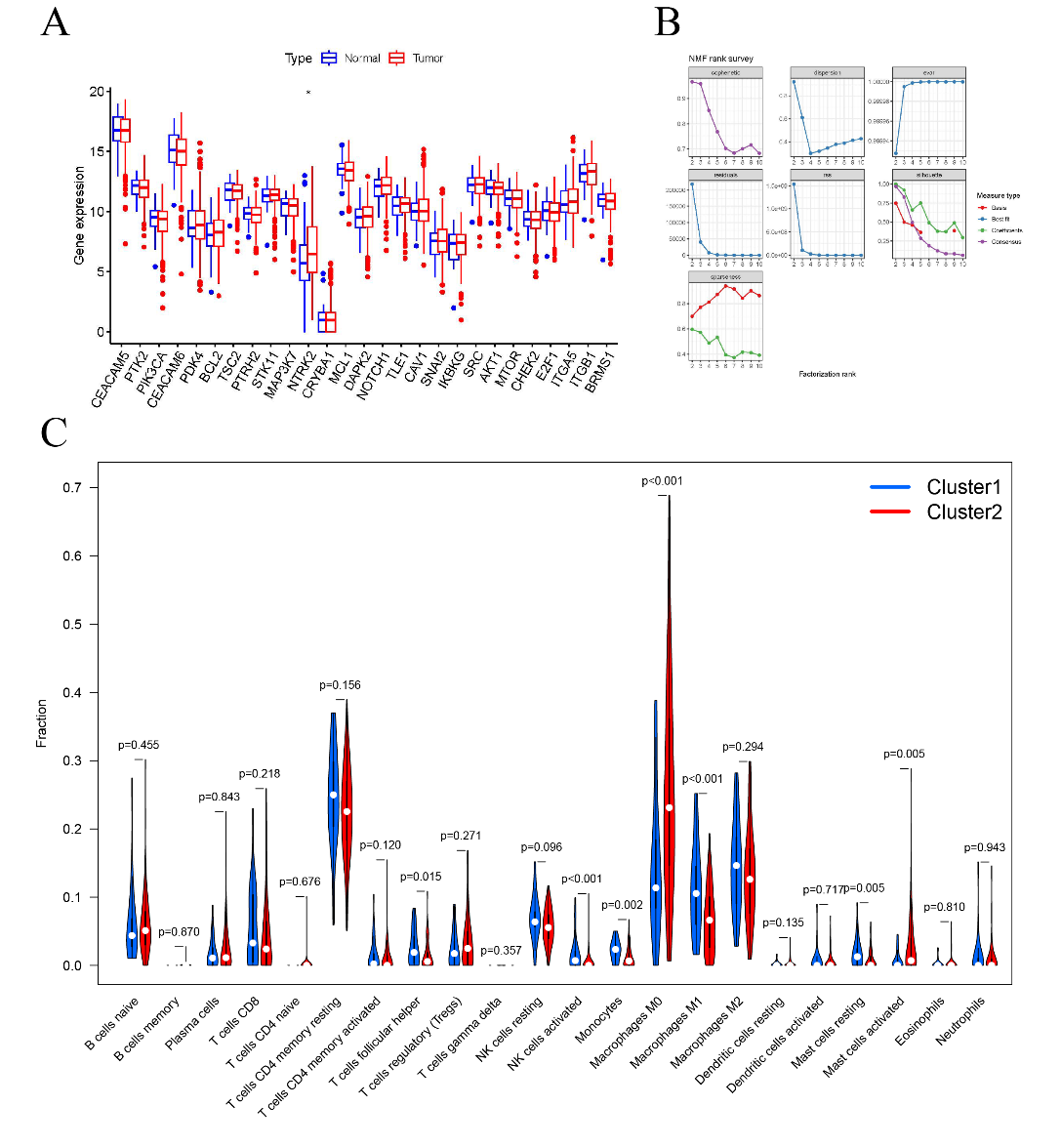


Supplementary Figure 1. (A) Basic mRNA expression of 27 ARGs. (B) NMF clustering. (C) Immune cell infiltration of two clusters.


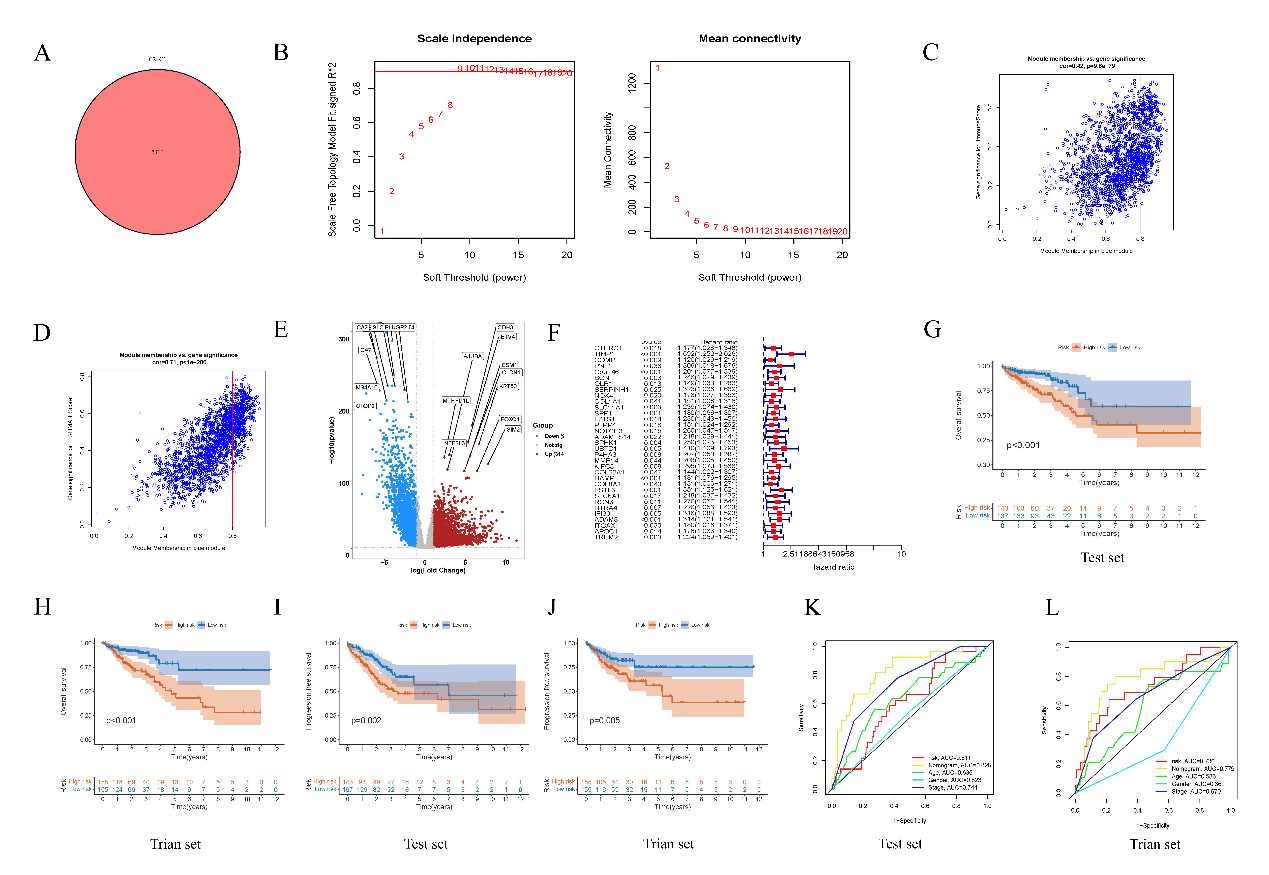


Supplementary Figure 2. WGCNA and risk model construction. (A) Total of 5314 CRC genes. (B) The soft threshold power for WGCNA. (C-D) The relationships with the immunescore and estimatescore. (E) Volcanic plot map of differential expression genes. (F) univariate analysis results of 79 genes. (G-J) KM curve of OS and PFS in test-set and train-set. (K-L) ROC curve of test-set and train-set.


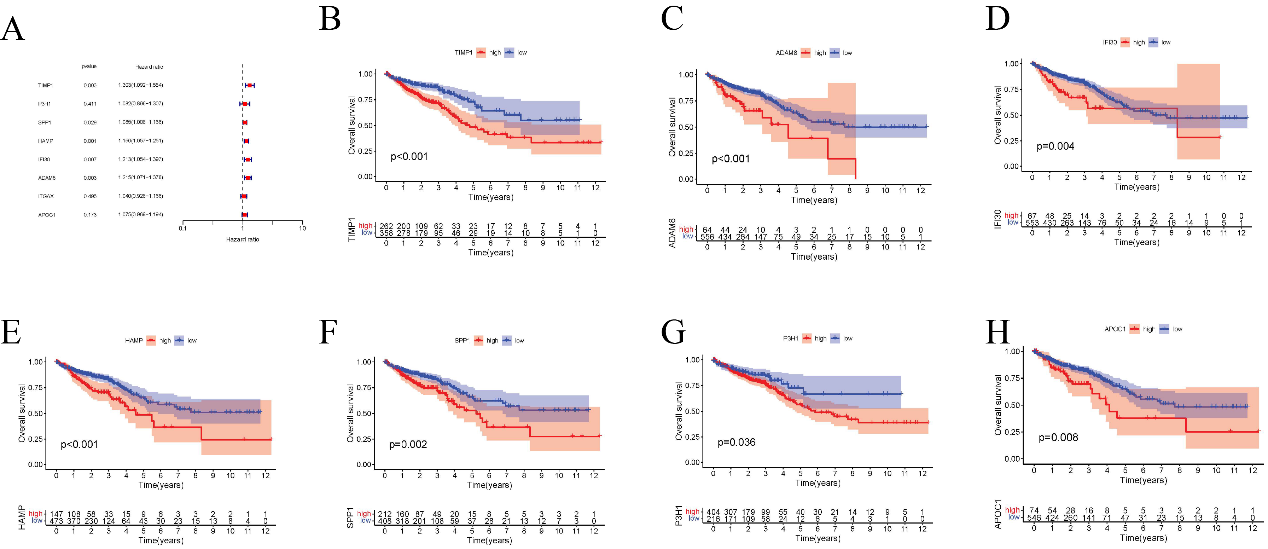


Supplementary Figure 3. Prognosis analysis of eight genes. (A) Univariate cox regression analysis (B-H) KM curve of those eight genes.


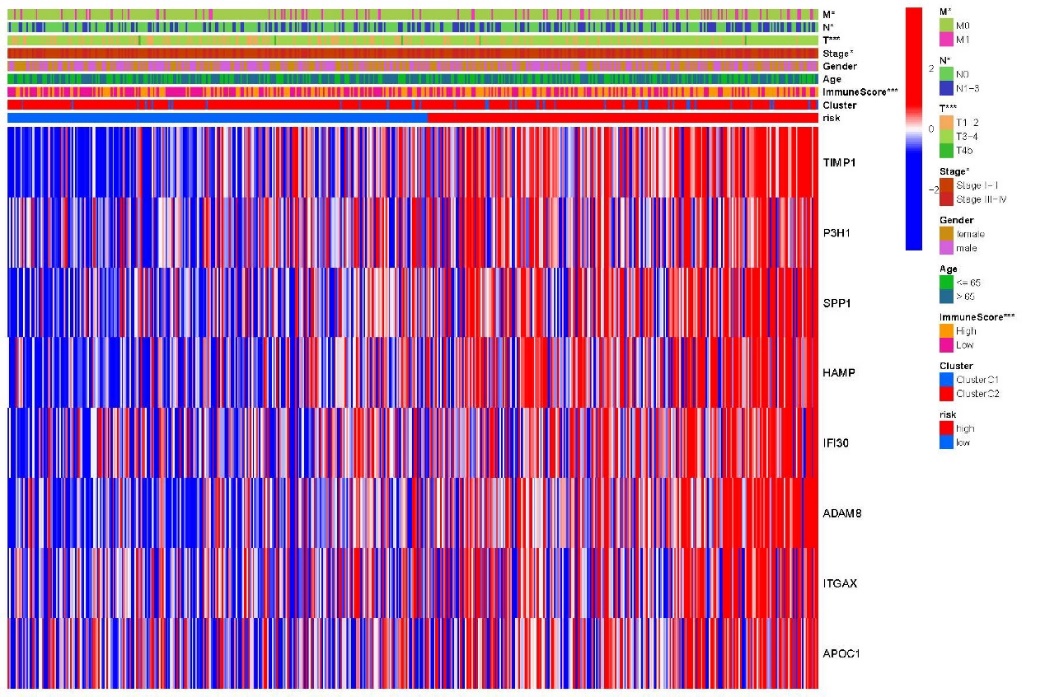


Supplementary Figure 4. Heatmap of the relationship between clusters, clinical traits, risk score and eight genes.


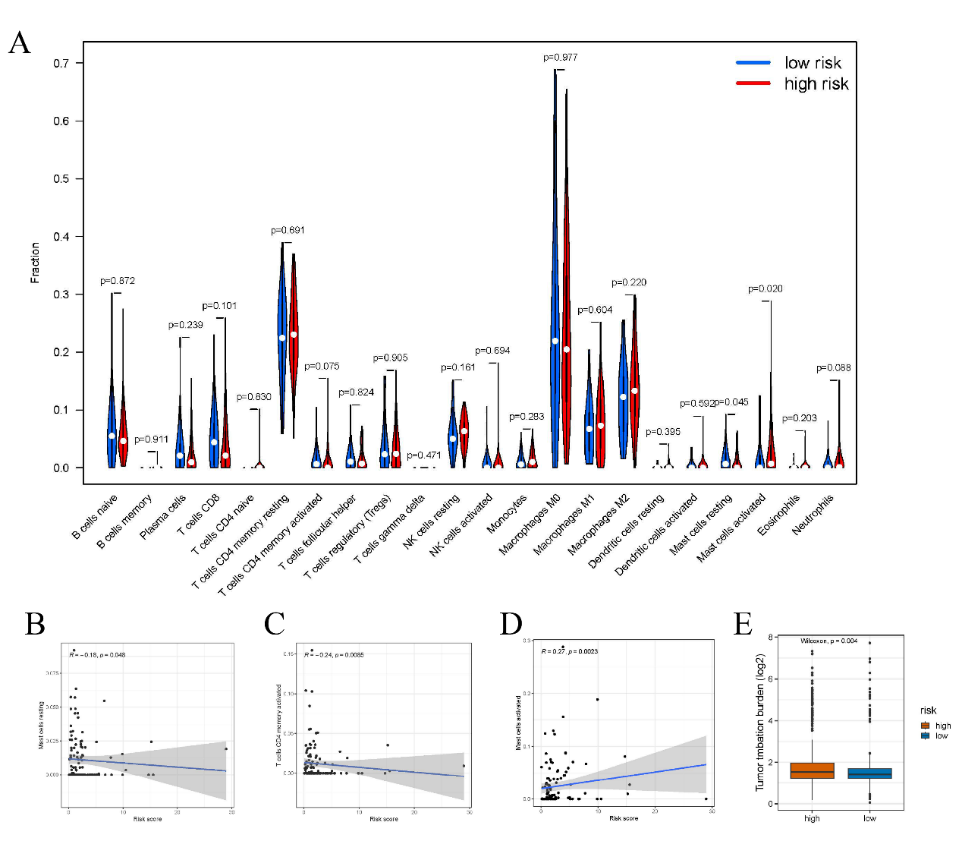


Supplementary Figure 5. Immune landscape analysis (A) Immune cell infiltration of two risk score group. (B-D) Risk score was negative correlation with mast cells resting and T cells CD4 memory activated, but positive correlation with mast cells activated. (E) TMB differences between two risk groups.


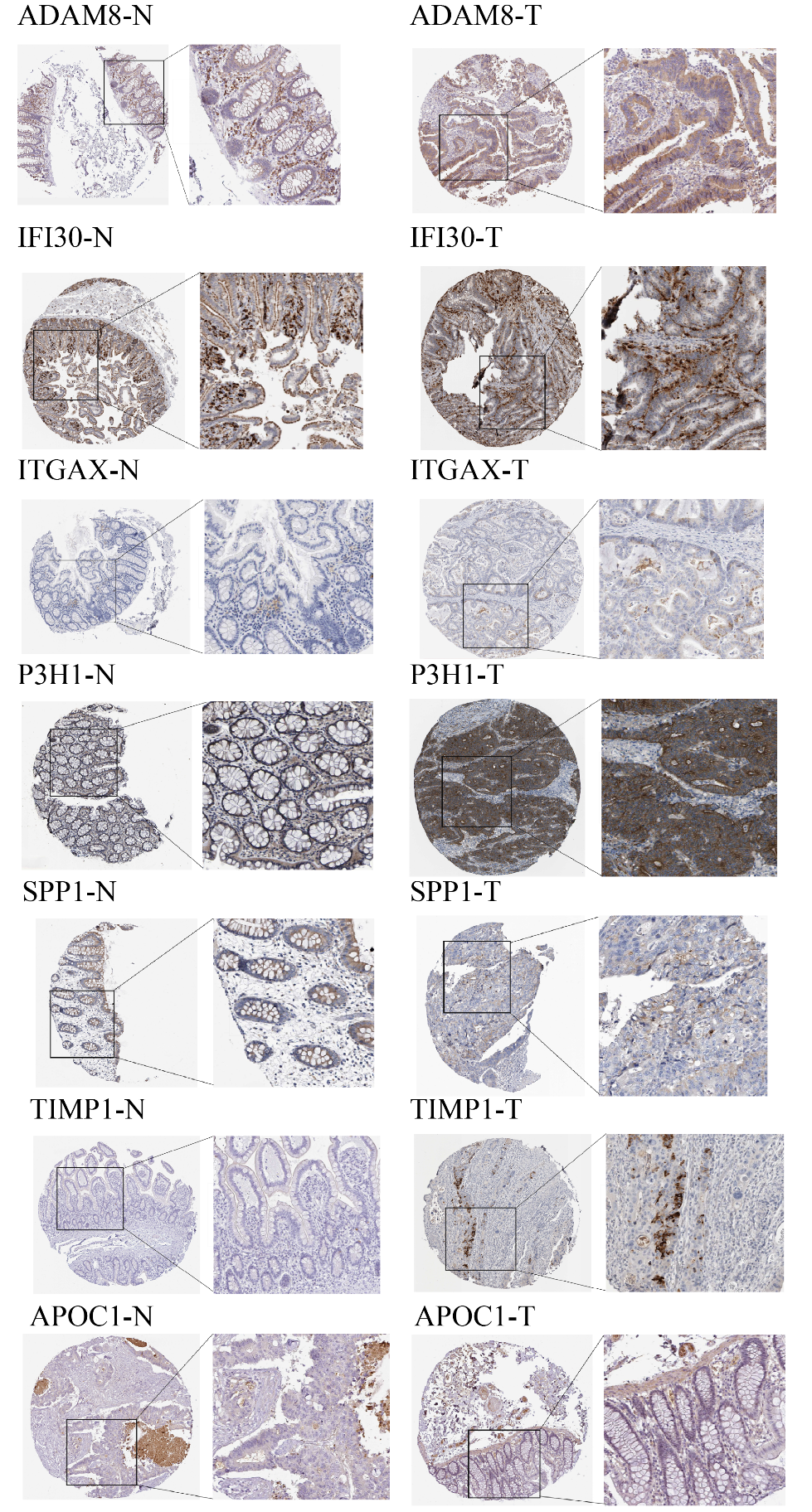


Supplementary Figure 6 Basic protein expression of eight genes except HAMP, N and T presented normal and tumour, respectively (magnification: ×2.5).

Supplementary Figure
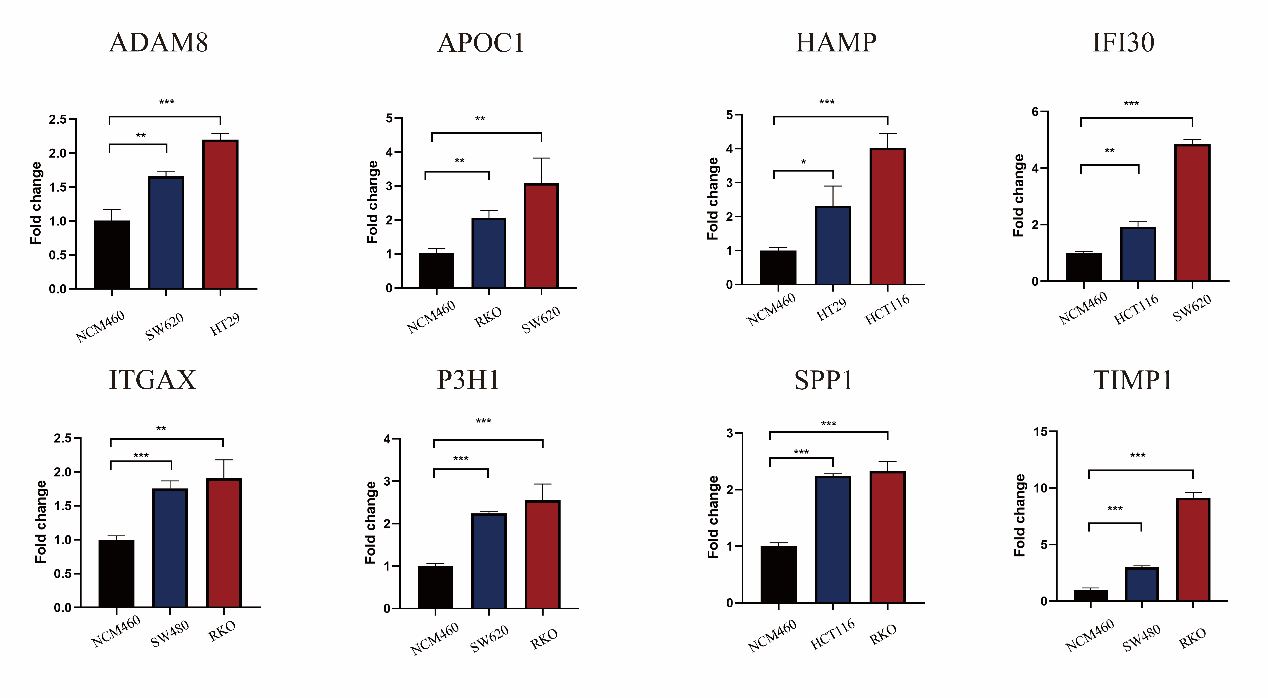
7 Hub gene expression validation in vitro.

Supplementary table 1. Primers of hub genes

| Gene | F | R |
| --- | --- | --- |
| TIMP1 | 5′-ATTCCGACCTCGTCATCAGG-3′ | 5′-GGACCTGTGGAAGTATCCGC-3′ |
| SPP1 | 5′-CTCCATTGACTCGAACGACTC-3′ | 5′-CAGGTCTGCGAAACTTCTTAGAT-3′ |
| HAMP | 5′-CTGACCAGTGGCTCTGTTTTC-3′ | 5′-GAAGTGGGTGTCTCGCCTC-3′ |
| IFI30 | 5′-CCCCTCTGCAAGCGTTAGAC-3′ | 5′-CCCGCAGGTATAGATTGCCT-3′ |
| ADAM8 | 5′-GAGGGTGAGCTACGTCCTTG-3′ | 5′-CAGCCGTATAGGTCTCTGTGT-3′ |
| ITGAX | 5′-AGAGCTGTGATAAGCCAGTTCC-3′ | 5′-AATTCCTCGAAAGTGAAGTGTGT-3′ |
| APOC1 | 5′-TCCAGTGCCTTGGATAAGCTG-3′ | 5′-GGCTGATGAGTTCCCGAGC-3′ |
| P3H1 | 5′-CACAGCGAGCGGGACAG-3′ | 5′-GCTCATCCTTGGGCTTCGAT-3′ |
| GAPDH | 5′-ATGACCACAGTCCATGCCAT-3′ | 5′-AAGGCCATGCCAGTG AGCTTC-3′ |

Supplementary table2. Clinical traits of CRC patients

| Traits | Type | Total | Test | Train | p-value |
| --- | --- | --- | --- | --- | --- |
| Age | ≤65 | 270(43.55%) | 136(43.87%) | 134(43.23%) | 0.9354 |
|  | >65 | 350(56.45%) | 174(56.13%) | 176(56.77%) |  |
| Gender | female | 291(46.94%) | 151(48.71%) | 140(45.16%) | 0.421 |
|  | male | 329(53.06%) | 159(51.29%) | 170(54.84%) |  |
| Stage | I | 105(16.94%) | 49(15.81%) | 56(18.06%) | 0.3168 |
|  | II | 227(36.61%) | 116(37.42%) | 111(35.81%) |  |
|  | III | 179(28.87%) | 85(27.42%) | 94(30.32%) |  |
|  | IV | 89(14.35%) | 52(16.77%) | 37(11.94%) |  |
|  | unknown | 20(3.23%) | 8(2.58%) | 12(3.87%) |  |
| T | T1 | 20(3.23%) | 8(2.58%) | 12(3.87%) | 0.4723 |
|  | T2 | 105(16.94%) | 50(16.13%) | 55(17.74%) |  |
|  | T3 | 423(68.23%) | 221(71.29%) | 202(65.16%) |  |
|  | T4 | 60(9.68%) | 25(8.06%) | 35(11.29%) |  |
|  | T4b | 10(1.61%) | 5(1.61%) | 5(1.61%) |  |
|  | Tis | 1(0.16%) | 1(0.32%) | 0(0%) |  |
|  | unknown | 1(0.16%) | 0(0%) | 1(0.32%) |  |
| N | N0 | 351(56.61%) | 175(56.45%) | 176(56.77%) | 0.385 |
|  | N1 | 150(24.19%) | 70(22.58%) | 80(25.81%) |  |
|  | N2 | 116(18.71%) | 64(20.65%) | 52(16.77%) |  |
|  | unknown | 3(0.48%) | 1(0.32%) | 2(0.65%) |  |
| M | M0 | 459(74.03%) | 230(74.19%) | 229(73.87%) | 0.2178 |
|  | M1 | 88(14.19%) | 51(16.45%) | 37(11.94%) |  |
|  | unknown | 73(11.77%) | 29(9.35%) | 44(14.19%) |  |

Supplementary table3. Clinical traits of clusters

|  | Clinical traits | C1 | C2 | P value |
| --- | --- | --- | --- | --- |
| n |  | 39 | 480 |  |
| Age (%) | <= 65 | 16 (41.0) | 215 (44.8) | 0.774 |
|  | > 65 | 23 (59.0) | 265 (55.2) |  |
| Gender (%) | female | 23 (59.0) | 223 (46.5) | 0.181 |
|  | male | 16 (41.0) | 257 (53.5) |  |
| Stage (%) | Stage I-II | 24 (61.5) | 271 (56.5) | 0.654 |
|  | Stage III-IV | 15 (38.5) | 209 (43.5) |  |
| T (%) | T1-2 | 4 (10.3) | 103 (21.5) | 0.193 |
|  | T3-4 | 35 (89.7) | 372 (77.5) |  |
| N (%) | N0 | 24 (61.5) | 281 (58.5) | 0.844 |
|  | N1-3 | 15 (38.5) | 199 (41.5) |  |
| M (%) | M0 | 33 (84.6) | 401 (83.5) | 1 |
|  | M1 | 6 (15.4) | 79 (16.5) |  |

Supplementary table4. Cox results of eight genes in the risk model

| ID | HR | HR.95L | HR.95H | p-value |
| --- | --- | --- | --- | --- |
| TIMP1 | 1.302922 | 1.092107 | 1.554431 | 0.003299 |
| P3H1 | 1.082258 | 0.896369 | 1.306695 | 0.410997 |
| SPP1 | 1.084575 | 1.008424 | 1.166475 | 0.028829 |
| HAMP | 1.149557 | 1.05673 | 1.25053 | 0.001176 |
| IFI30 | 1.213417 | 1.053945 | 1.397018 | 0.007128 |
| ADAM8 | 1.21464 | 1.070695 | 1.377937 | 0.002517 |
| ITGAX | 1.04049 | 0.928321 | 1.166213 | 0.495243 |
| APOC1 | 1.075425 | 0.968526 | 1.194123 | 0.173425 |

Table 5. Clinical traits of six CRC patients

| Gender | Age(year) | Site | T | N | M |
| --- | --- | --- | --- | --- | --- |
| Female | 79 | Rectum | 2 | 0 | 0 |
| Male | 60 | Rectum | 3 | 0 | 0 |
| Female | 74 | Rectum | 3 | 0 | 0 |
| Female | 67 | Rectum | 4 | 0 | 0 |
| Female | 74 | Colon | 2 | 0 | 0 |
| Male | 63 | Colon | 1 | 0 | 0 |
